# Differentiate or Die: 3-Bromopyruvate and Pluripotency in Mouse Embryonic Stem Cells

**DOI:** 10.1371/journal.pone.0135617

**Published:** 2015-08-12

**Authors:** Ana Sofia Rodrigues, Sandro L. Pereira, Marcelo Correia, Andreia Gomes, Tânia Perestrelo, João Ramalho-Santos

**Affiliations:** 1 PhD Programme in Experimental Biology and Biomedicine, CNC—Center for Neuroscience and Cell Biology, University of Coimbra, Coimbra, Portugal; 2 CNC—Center for Neuroscience and Cell Biology, University of Coimbra, Coimbra, Portugal; 3 Institute for Interdisciplinary Research (IIIUC), University of Coimbra, Coimbra, Portugal; 4 Biocant—Center of Innovation in Biotechnology, Cantanhede, Portugal; 5 Department of Life Sciences, University of Coimbra, Coimbra, Portugal; University of Kansas Medical Center, UNITED STATES

## Abstract

**Background:**

Pluripotent embryonic stem cells grown under standard conditions (ESC) have a markedly glycolytic profile, which is shared with many different types of cancer cells. Thus, some therapeutic strategies suggest that pharmacologically shifting cancer cells towards an oxidative phenotype, using glycolysis inhibitors, may reduce cancer aggressiveness. Given the metabolic parallels between cancer and stemness would chemotherapeutical agents have an effect on pluripotency, and could a strategy involving these agents be envisioned to modulate stem cell fate in an accessible manner? In this manuscript we attempted to determine the effects of 3-bromopyruvate (3BrP) in pluripotency. Although it has other intracellular targets, this compound is a potent inhibitor of glycolysis enzymes thought to be important to maintain a glycolytic profile. The goal was also to determine if we could contribute towards a pharmacologically accessible metabolic strategy to influence cell differentiation.

**Methodology/Principal Findings:**

Mouse embryonic stem cells (mESC) grown under standard pluripotency conditions (in the presence of Leukemia Inducing Factor- LIF) were treated with 3BrP. As a positive control for differentiation other mESCs were grown without LIF. Overall our results demonstrate that 3BrP negatively affects pluripotency, forcing cells to become less glycolytic and with more active mitochondria. These changes in metabolism are correlated with increased differentiation, even under pluripotency conditions (i.e. in the presence of LIF). However, 3BrP also significantly impaired cell function, and may have other roles besides affecting the metabolic profile of mESCs.

**Conclusions/Findings:**

Treatment of mESCs with 3BrP triggered a metabolic switch and loss of pluripotency, even in the presence of LIF. Interestingly, the positive control for differentiation allowed for a distinction between 3BrP effects and changes associated with spontaneous differentiation/loss of pluripotency in the absence of LIF. Additionally, there was a slight differentiation bias towards mesoderm in the presence of 3BrP. However, the side effects on cellular function suggest that the use of this drug is probably not adequate to efficiently push cells towards specific differentiation fates.

## Introduction

Embryonic stem cells (ESC) rely more on glycolysis and have few immature mitochondria, localized mainly around the nucleus [1–3]. Furthermore, although there may be a metabolically bivalent metabolic state early in cell commitment a shift from glycolysis to a predominantly oxidative metabolism (OXPHOS) is needed for differentiation to take place [4–6]. Indeed, low O_2_ tension and “silent”/quiescent mitochondria are beneficial for pluripotency, which is also boosted by mitochondrial inhibition [7, 8]. Moreover, the activation of the internal pluripotency network in induced pluripotent stem cells (iPSC) during somatic cell reprogramming is preceded by a prior metabolic shift towards glycolysis [9], and the modulation of the pentose phosphate pathway leads to a biased differentiation [10].

Importantly, the metabolic characteristics of pluripotent stem cells (PSCs) are common to proliferative cells in general, and thus similar to some types of cancer cells. Common metabolic strategies between cancer and stemness include high levels of hexokinase II (HKII) linked to the outer mitochondrial membrane and a pyruvate dehydrogenase (PDH) cycle promoting the conversion of pyruvate to lactate rather than to acetyl-CoA [11]. Hexokinase is a key glycolytic enzyme that phosphorylates glucose to glucose 6-phosphate (G-6-P), and thus trapping it inside the cell. Certain tumor cells upregulate HKII expression due to its higher affinity for glucose and its privileged location in the outer mitochondrial membrane [12]. Depletion of HKII in tumor cells increases sensitivity to cell death [13] and HKII inhibits aerobic glycolysis, leading to an increase in OXPHOS [14]. Of course other key metabolic players should be considered, such as Hypoxia inducible factor-1alpha (HIF-1a) and c-Myc [7, 15–17]. In fact, tumor aggressiveness and progression have been shown to positively correlate with a hypoxic microenvironment due to a high activity of HIF-1a and c-Myc [18, 19] enhancing the transcription of genes coding for glycolytic enzymes and other important signaling pathways that help promote aerobic glycolysis, or the Warburg effect [15, 17, 20].

Taken together these data suggest that pharmacological strategies linked to the targeting of metabolic characteristics that define active cancer cells may also be useful in modulating pluripotent stem cell fate. Although it may also have other targets, 3-brompyruvate (3BrP) is a chemical pyruvate analog that functions as a potent inhibitor of glycolytic enzymes, most notably, but not exclusively, HKII [21, 22]. Importantly 3BrP has been used as an anti-cancer drug, including in clinical trials, and shown to induce cell death and thus reducing tumor size [2, 23, 24]. However, the exact mechanism by which this compound acts as an anti-tumor drug, is not completely known, although 3BrP treatment alters mitochondrial function in terms of both reactive oxygen species (ROS) and ATP production [25].

Given that ESCs are also highly proliferative, and share some common features with tumor cells, we wondered if 3BrP would also affect ESC pluripotency and could, in addition, be used as a practical and inexpensive tool to modulate stem cell fate by promoting differentiation by inducing a metabolic glycolysis-OXPHOS shift. The goal of the present work was not to determine the importance of specific glycolysis enzymes towards the maintenance of pluripotency, for which a totally different experimental approach would be necessary, but rather to establish if the specific compound 3BrP could be a viable alternative for cell culture metabolic modulation in order to obtain specific cell lineages.

## Material and Methods

### Cell culture conditions and Experimental design for 3-Bromopyruvate (3BrP)

Mouse embryonic stem cell line E14Tg2a was kindly provided by Miguel Ramalho-Santos (University of California, San Francisco, USA) and characterized elsewhere [26, 27]. Cells were maintained in feeder free conditions using Knockout-DMEM media (Gibco Life Technologies) supplemented with 15% of KSR (Knockout serum replacement—Gibco LIFe Technologies), 1% of MEM Non-Essential Amino acids (Sigma-Aldrich), 1% Penicillin/Streptomycin, 1% L-glutamine (2mM) (both from Gibco LIFe Technologies) and β-Mercaptoethanol (Sigma-Aldrich). In order to maintain pluripotency Leukemia inhibitor factor (LIF; ESGRO Millipore) was added to a working concentration of 1000U/ml. Media was changed every 24h and cells were maintained at 37°C, 20%O_2_ and 5% CO_2_. E14Tg2a mESC were passaged when the right confluence was achieved, usually two or three days after platting. Briefly, 0.1% gelatin (Sigma-Aldrich) was added to plates and allowed to coat for ten minutes at 37°C. Afterwards, the excess was removed and supplemented Knockout-DMEM media was added. Cells were plated at a final density of 5000cells/cm for all experimental conditions: ESCs in control conditions (with LIF), in the absence of LIF (differentiation control) and in the presence of two different 3BrP (Sigma-Aldrich) concentrations (25 and 50 μM) plus LIF. 3BrP was freshly prepared and added every 24hours and experiments were conducted after 60h of incubation with 3BrP+LIF or in the absence of LIF. It is important to stress that all 3BrP experiments were carried out under strict pluripotency conditions (i.e. in the presence of LIF) in order to determine if this glycolytic inhibitor pushed the cells towards differentiation even under pluripotency conditions. Thus 3BrP experiments were compared to ESCs cultured with (pluripotency control) or without (differentiation control) LIF. The exception was the embryoid body experiments, specific for differentiation, as noted below.

### Viability

In order to monitor cell viability the LIVE/DEAD Kit (Invitrogen) was used according to manufacturers’ instructions. The Kit consists of two DNA binding fluorescent dyes: SYBR 14 which is membrane permeable staining the nucleus green for all cells, and PI (propidium iodide). PI only enters cells with compromised membrane integrity, thus staining the nucleus of dead cells red. Briefly, cells were collected at the 60h time point by enzymatic dissociation with accutase (Gibco LIFe Technologies), and centrifuged for 5 min at 1200rpm. The pellet was ressuspended in D-PBS and 6μM of SYBR 14 and 0.48mM of PI were added to the cell suspension that was then incubated for 20 min at 37°C, 20%O_2_ and 5% CO_2_. Viability was assessed counting 100 cells per condition; green fluorescent cells without red fluorescence were counted as live cells and a cell with both stains as dead cells using a fluorescent microscope.

### Thiazolyl Blue Tetrazolium Bromide (MTT) assay

MTT was reconstituted according to the manufacturers’ instructions (Sigma-Aldrich) and was used at a final concentration of 0.5mg/ml. This assay is routinely to monitor cell proliferation/metabolic activity. This is due to the fact that MTT is reduced by cellular dehydrogenases (using both NADPH and NADH) present in the cells, and this will produce violet formazan crystals that are soluble in acidified isopropanol [28]. Cells were plated and incubated for 60h and media was changed so that the assay did not take place in the presence of 3BrP. Formazan crystals (violet) formed after a 5 h incubation at 37°C, 20%O_2_ and 5% CO_2_ and were solubilized with 300 μl of isopropanol with 0.04M HCl. Intensity was measured colorimetrically at 570 nm. Raw data was normalized to total cell number for each condition and then metabolic activity was normalized to the control.

### Alkaline phosphatase (AP) assay

It is well accepted that stem cells that are self-renewing and pluripotent present high levels of AP [29]. The Alkaline Phosphatase Detection Kit from Millipore was used for each experimental condition following the manufacturers’ instructions. Briefly, cells were cultured in a 24 well plate for 60h, media was removed and cells were fixed with 4% paraformaldehyde for 1 min. Cells were washed and the alkaline phosphatase reagent (prepared according to the manufacturers’ instructions) was added. After a 20 min incubation at room temperature (RT) in the dark the reagent was removed and D-PBS was added. Colonies were counted using an optical microscope; red colonies were considered AP positive while unstained colonies were counted as AP negative. All colonies in the 24 wells were counted and counts normalized to 100%. Experiments were performed in duplicate for all experimental conditions.

### Flow cytometry

All dyes noted below were analyzed by flow cytometry (BD FACSCalibur) and 20000 gated cells were acquired/analyzed per condition with the Cell Quest Pro Acquisition software (BD Biosciences).

Mitochondrial membrane potential (MMP) was monitored using tetramethylrhodamine methyl (TMRM-Invitrogen) [30–32] a lipophilic cationic fluorescent dye that, due to its positive charge, accumulates in mitochondria according to membrane potential. Cells were incubated with 20μM of TMRM for 20 min at 37°C, 20%O_2_ and 5% CO_2_ in the dark in 1ml of D-PBS. Afterwards cells were centrifuged to remove excess TMRM and pellets were ressuspended with 500μl of PBS, kept on ice and analyzed. In order to define the proper gates for an accurate analysis we used cells without TMRM as a blank control and TMRM labeled cells incubated with 250 μM of CCCP, a potent mitochondrial uncoupler, as a negative control.

To evaluate intracellular amounts of superoxide anion we used MitoSOX Red (Molecular Probes) that emits fluorescence after selectively reacting with superoxide in mitochondria. MitoSOX Red was prepared according to the manufacturers’ instructions and cells were incubated for 30 min at 37°C in the dark with a final concentration of 3μM of the probe. To properly define the analysis gates we used cells without the probe as a negative control and cells that were incubated with Antimycin A (100μM) as a positive control, given that this potent mitochondrial complex III inhibitor acts as a ROS inducer [33–35].

To infer effects on cell proliferation we examined the expression of the proliferating cell nuclear antigen (PCNA), given its role in DNA replication and repair, and that it is highly expressed in rapidly proliferating cells [36]. Cells were fixed with 70% ethanol and stored overnight at -20°C, then subjected to an acidic denaturation step with 2N HCl and washed. Afterwards cells were incubated for 1h with primary antibody against PCNA (1:100), washed with D-PBS and FITC-conjugated secondary antibody was added (1:100) for 1h in the dark [37]. As negative controls we used cells without antibodies (to access auto-fluorescence), as well as cells incubated only with the primary antibody and cells incubated only with the secondary antibody.

For all flow cytometry experiments representative data is also available in S1 Fig.

### Adenine nucleotide content analysis by High Performance liquid chromatography (HPLC)

The protocol for adenine nucleotide content analysis was as previously described [11]. Samples were stored at -80°C until assayed by separation in a reverse-phase HPLC using a Beckman-System Gold. The detection wavelength was 254 nm, and the column used was a LiChrospher 100 RP-18 (5 μM, Merck). The elution buffer was composed by 100mM phosphate buffer (pH 6.5) and supplemented with 1% methanol. Retention times were determined using standards for ATP, ADP and AMP (Sigma-Aldrich): Adenylate Energy Charge was calculated according to the following formula: ATP+0.5xADP/(ATP+ADP+AMP) [38, 39].

### Total RNA isolation, DNA cleanup, cDNA synthesis and RT-PCR

RNA isolation and DNA cleanup was performed as described previously [40]. RNA concentration and quality were determined using NanoDrop 2000 (Thermo Scientific) and samples presenting a 260/280 ratio under 1.8 were discarded. Samples of total RNA were stored at -80°C until use [40]. cDNA was obtained using the iScript cDNA Synthesis Kit from Bio Rad according to the protocol established by the manufacturer. Samples were then placed in the thermal cycler (S1000 Thermal Cycler) programmed with a reaction protocol provided by the manufacturer. RT-PCR was performed to quantify gene expression for OCT4; NANOG; GAPDH; HEXOKINASEII AND HEXOKINASEI with BETA-Actin used as housekeeping gene for data normalization. Primers are described in S1 Table and were obtained from a primer bank database (http://pga.mgh.harvard.edu/primerbank/) and ordered from Integrated DNA Technologies (IDT). SsoFast EvaGreen Supermix (Bio-Rad) was used to perform RT-PCR analysis according with Bio-Rad instructions. Samples were run in CFX96 Touch Real-Time PCR Detection System and mRNA fold change was calculated using the -ΔΔCt method.

### Total Protein Extracts, protein quantification and Western Blot

In order to obtain protein extracts for Western blot analysis mESC were lysed with 100μl of RIPA buffer (Sigma-Aldrich) supplemented with 2mM of phenylmethylsulphonyl fluoride-PMSF (Sigma-Aldrich) and 2x Halt phosphatase inhibitor cocktail (Pierce, Rockford, IL) as described elsewhere [11]. Protein quantification was performed using the Pierce BCA (Bicinchoninic Acid) Protein Assay Kit, following the datasheet protocol. Both samples and calibration curve were determined with duplicates.

Protein samples for Western blot were prepared by diluting 30μg of protein in Laemmli sample buffer (Bio-Rad) and water, given that the volume of sample buffer had to contain 30μg of protein (water was used to adjust the volume). A total volume of 30μl per sample was prepared and denatured at 95°C in a dry bath. After this step samples were loaded into 12% Acrilamide Tris-HCl gel and electrophoresis was performed in a Mini protean tetra cell Bio-Rad apparatus. After protein size separation in the gel, proteins were blotted into a PDVF membrane (Bio-Rad) and blocked in a solution of 5% powder milk (Bio-Rad) in Tris-Buffered Saline with Tween (TBST). Afterwards, membranes were incubated overnight at 4°C with the primary antibodies: mouse anti-Nanog (Santa Cruz Biotechnology, 1:500), rabbit anti-Oct4, mouse anti-P53, rabbit anti-PDH, rabbit anti-GAPDH (Cell Signaling Technology, 1:2000), mouse anti-Hexokinase I, mouse anti-Hexokinase II, mouse anti-Caspase3 (Cell Signaling Technology, 1:500) and mouse anti-beta-Actin (Sigma-Aldrich, 1:5000). Membranes were washed and then incubated with the correct HRP-conjugated secondary antibody. Proteins were detected using the Clarity Western ECL Substrate (Bio-Rad) and membranes were developed using the VersaDoc Imaging system (Bio-Rad). Protein quantification was performed using Quantity One software and results were normalized to beta-Actin levels for each condition.

### Embryoid Body (EB) formation

To promote and monitor ESC differentiation the EB assay was used. mESCs were platted in a 60mm non-adherent petri dish at a cellular density equal to one million cells and were left for three days in a 37°C, 5% CO2 incubator with KODMEM without LIF supplementation in order to form EB. Cell media was changed during this period by gentle aspiration of the non-adherent dishes into falcons and allowing the cells to sediment. At day 3 KODMEM media was supplemented with 10% FBS and EB were transferred into new normal gelatin coated 100mm dishes. EBs were incubated overnight in a 37°C, 5% CO2 incubator to allow them to attach to the dish. From that day on EB were grown with basic DMEM media with no serum supplementation and media changed every other day. At day 14 EBs were collected for protein isolation. In this case 3BrP experiments were carried out in the absence of LIF, as the goals was to see if the drug created a differentiation bias. Therefore there is only one control in this case (EB formation without 3BrP), unlike what took place in the pluripotency experiments mentioned earlier.

### Statistical Analysis

SPSS Statistics 21.0 (SPSS Inc.) was used to perform statistical analysis. All the raw data was verified for normality and homoscedasticity and accordingly the appropriate parametric tests for paired samples were applied. Given that all the experimental conditions were collected/analyzed at the same time a repeated measure ANOVA was used. Sphericity was assessed and, when violated, the Greenhouse-Geisser correction was applied before performing any POST-HOC tests. When data violated the normal distribution assumption a Friedman ANOVA was performed. All data is expressed as mean ± standard error of mean (SEM) reflecting the number of experiments. Statistical significance was determined at p ≤ 0.05.

## Results

### Effect of 3BrP on cell number, morphology, viability and pluripotency

**Fig 1:**
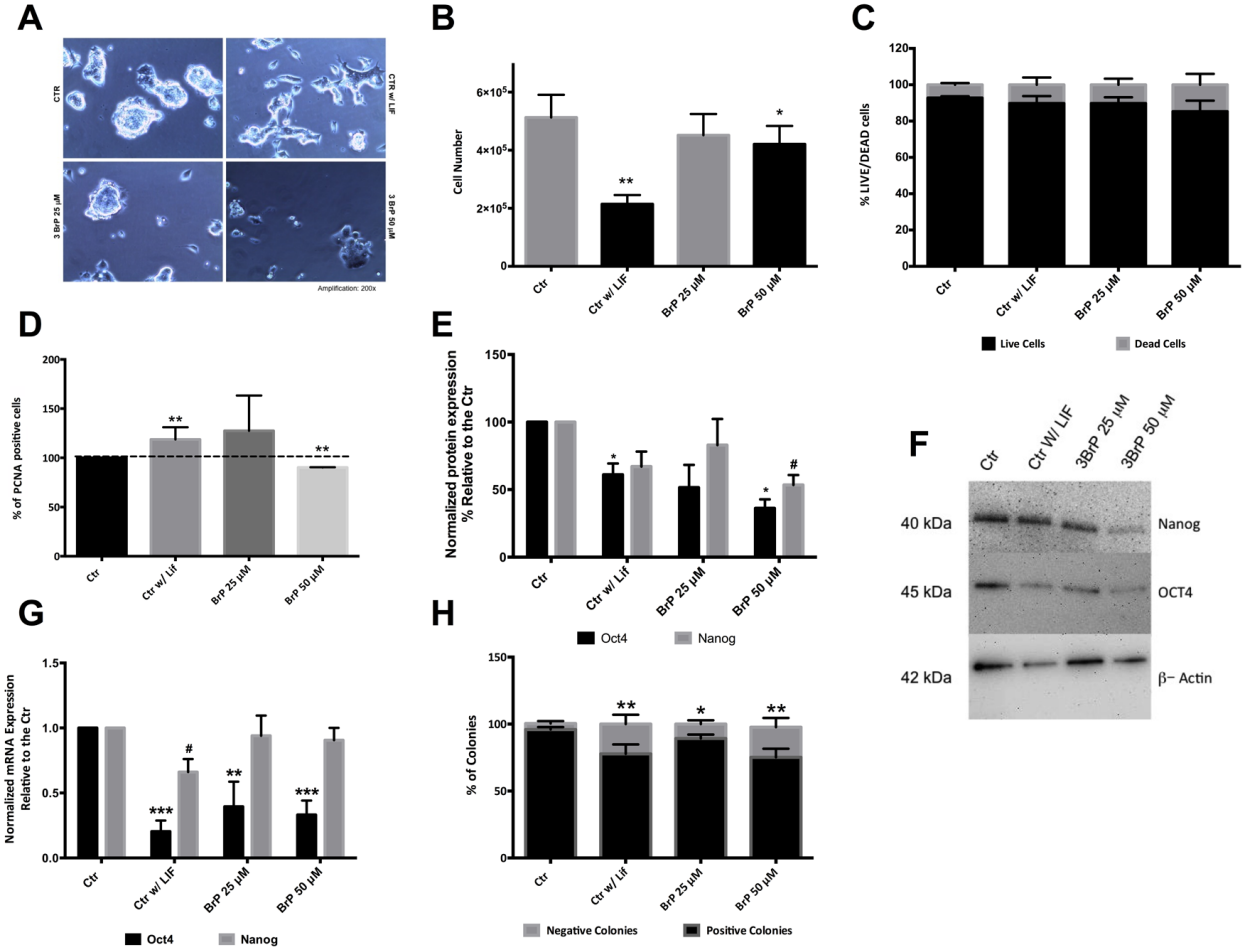
Effect of 3BrP on morphology, cell number, viability, proliferation and pluripotency. ESCs were maintained in control conditions (with LIF), in the absence of LIF (differentiation control) and in the presence of two different 3BrP concentrations (25 and 50 μM) plus LIF. **A)** Phase microscopy photographs of the colonies in all conditions with a magnification of 200x. **B)** Total number of cells counted upon collection for ATP and flow cytometry experiments. **C)** Percentage of viable *versus* dead cells. Cells stained green were counted as live, while PI positive cells were counted as dead. 100 cells were counted per condition for a total of n = 8 independent experiments, results are expressed as means ± SEM for 30 experiments. **D)**- Cells in all experimental conditions were stained for PCNA in the cell nucleus and protein levels were assessed by flow cytometry. Results were analyzed in terms of Geometric Mean of Fluorescence for 20000 cells for each condition and are represented as percentage relative to the control; 4 independent experiments were performed. **E)**- Western Blot quantification by densiometric evaluation for Oct4 and Nanog. Results are presented as percentage relative to the CTR and were normalized for beta-actin; 5 independent experiments were performed. **F)**- Representative blot demonstrating the decrease in protein levels for pluripotency markers. **G)**- RT-PCR analysis for the Oct4 and Nanog mRNA gene levels are represented as fold changes normalized to the Control after normalization for endogenous beta-actin; 4 independent experiments were performed. Results are expressed as means ± SEM. **H)**- Quantification for the alkaline phosphatase assay: colonies stained red were counted as positive (pluripotent) colonies whereas unstained colonies were counted as negative. A total of 10 independent experiments were analyzed and results are expressed as means ± SEM. *p≤ 0.05, **p≤ 0.01, ***p≤ 0.001.

Given that 3BrP has been described as lowering cell viability by increasing ROS production and activating the mitochondrial apoptosis pathway in glycolytic cells [13, 41], we started by analyzing possible 3BrP effects in mESC total cell number, morphology and viability. While in control conditions bright tightly packed round colonies growing as a dome could be seen, cells growing without the essential pluripotency factor leukemia inhibitor factor (LIF; as a negative control for pluripotency/self-renewal) no longer contained characteristic pluripotent colonies. Instead, higher levels of differentiation were obvious, resulting in less bright colonies without defined borders. On the other hand, cells grown with LIF but in the presence of 3BrP, while retaining well-defined round colonies, also showed increased differentiation in comparison with the control (Fig 1A). However, cultures treated with the higher concentration of 3BrP also had lower total cell numbers when compared to controls although this effect was even more visible in cells grown without LIF (Fig 1B), suggesting that, while 3BrP impacts cell number in the presence of LIF, the absence of LIF had a more pronounced, and deleterious, effect. Interestingly, results with the LIVE/DEAD Kit show that, although there are fewer cells for some experimental conditions, these cells are alive (Fig 1C), and cell viability is therefore not responsible for the decrease in cell numbers. We explored the possibility that 3BrP could be disrupting mESC proliferation by evaluating Proliferating Cell Nuclear Antigen (PCNA) levels by flow cytometry (Fig 1D). Results for 50 μM 3BrP showed a significant decrease in cell proliferation (p<0.01), which could explain the decrease in cell number, and the fact that colonies are smaller under these conditions. However, and in contrast, an increase in PCNA levels was seen in the negative control (p<0.01).

Does 3BrP affect pluripotency in the presence of LIF? To address this issue pluripotency was first monitored by analyzing gene expression and protein levels for two of the major regulators of the pluripotency network: Oct4 and Nanog. Both Western Blot (Fig 1E and 1F) and RT-PCR (Fig 1G) show a tendency for a negative effect of 3BrP on pluripotency in the presence of LIF, notably for Oct4. At the protein level the most deleterious effect occurred for 50 μM 3BrP where both Oct4 and Nanog levels were significantly decreased (p<0.05). Interestingly, the 3BrP-induced negative effect on pluripotency is greater in terms of mRNA levels for Oct4. As expected, our negative control (cells grown without LIF) had decreased protein and mRNA levels for both pluripotency markers confirming compromised pluripotency. Given that pluripotent cells have high levels of Alkaline Phosphatase (AP), we also performed the AP assay which monitors the number of AP-positive red colonies [29, 42] (Fig 1H) As expected controls had a high percentage of AP-positive colonies (over 90%), while cells growing without LIF had a lower percentage of AP-positive colonies (P<0.01). Interestingly, we observe a similar decrease for cells treated with 3BrP in the presence of LIF. Overall the results clearly suggest a deleterious effect of 3BrP on pluripotency, similar to the effect promoted by removing LIF.

### Mitochondrial function and Energy status of mESCs in the presence of 3BrP

**Fig 2:**
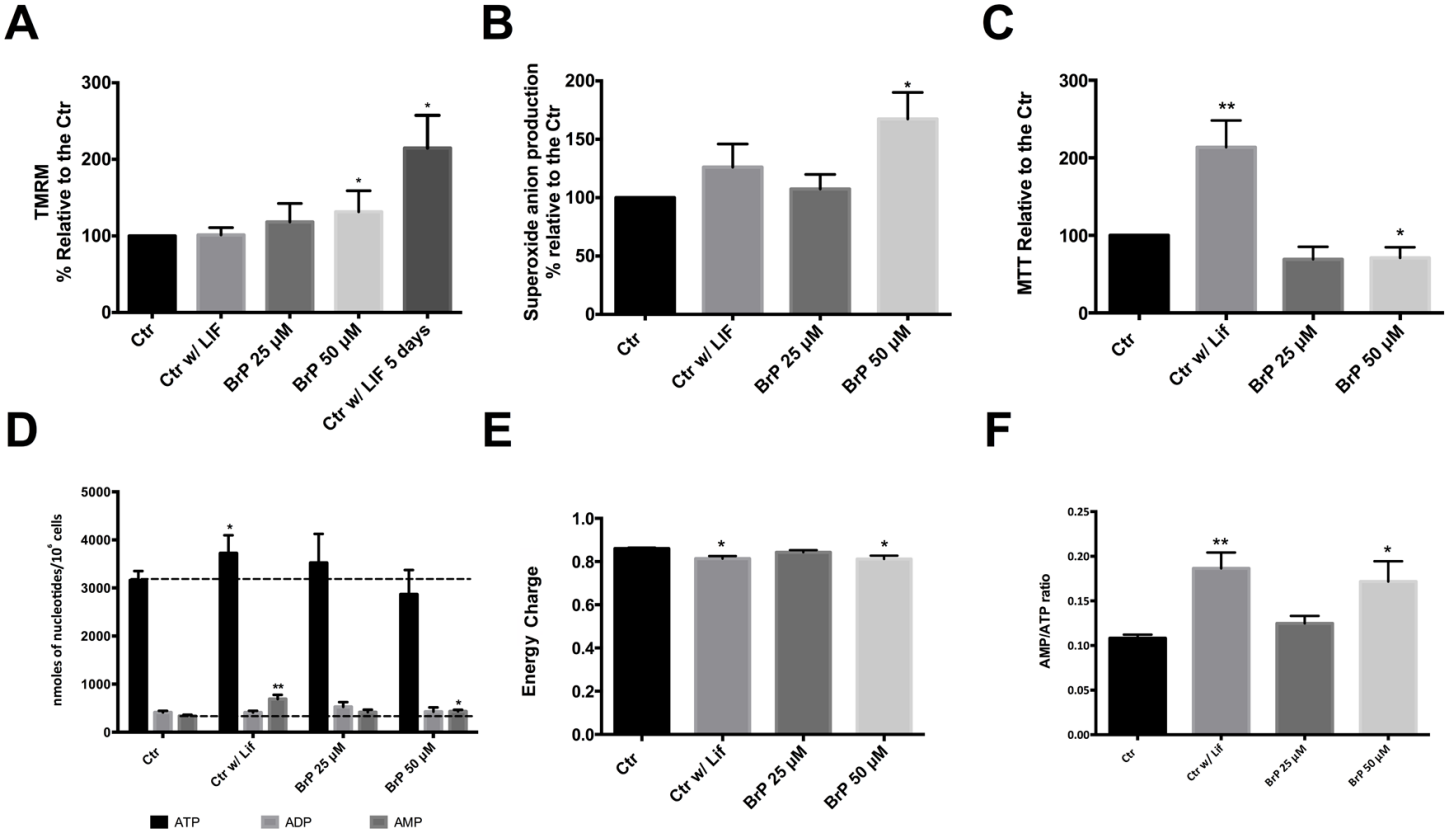
Assessing 3BrP effects on mitochondrial function. ESCs were maintained in control conditions (with LIF), in the absence of LIF (differentiation control) and in the presence of two different 3BrP concentrations (25 and 50 μM) plus LIF. For each flow cytometry experiment results were analyzed in terms of Geometric Mean of Fluorescence for 20000 cells for each condition and are represented as percentage relative to the control. **A)**- Quantification of mitochondrial membrane potential (MMP) potential using TMRM; 4 independent experiments were performed. **B)**- Quantification of superoxide production using MitoSOX Red dye; 4 independent experiments performed. See also S1 Fig. **C)**- Results for the MTT assay are presented as percentage of formazan crystals relative to the control. Because the goal was to evaluate oxidative status MTT results were normalized for total cell number. A total of 10 independent experiments were performed. *p≤ 0.05, **p≤ 0.01. **D)**- Nucleotide levels determined by HPLC in 10 independent experiments. **E)**- Energy charge calculated as ([ATP] + 0.5×[ADP])/([ATP] + [ADP] + [AMP]). **F)**- AMP/ATP ratio n = 10.

Given that 3BrP has been described to cause mitochondrial depolarization [43] and that and differentiated cells should have a higher mitochondrial membrane potential (MMP)[44] we assessed mitochondrial membrane potential with the Tetramethylrhodamine, methyl ester (TMRM) dye. Interestingly, our results show a trend towards an increase in MMP in the presence of 3BrP (Fig 2A). Taking into account that, while it has been described that with differentiation mitochondria become more polarized, our negative control (absence of LIF for 60 h) was not confirming this trend, we cultured cells in the absence of LIF for 5 days, and this indeed resulted in a significant increase in mitochondrial membrane potential.

Mitochondrial ROS production, in the form of mitochondria-produced superoxide anion, was also analyzed as another indicator of mitochondrial activity and possible target for 3BrP induced changes in ESC status. Superoxide anion production was quantified by measuring MitoSOX Red fluorescence by flow cytometry, and showed a similar trend as noted for TMRM; all the experimental conditions presented a slight increase in superoxide anion production when compared to the control, although this increase was statistically significant for 50 μM 3BrP (Fig 2B). This is in accordance with previous literature describing that 3BrP exposure causes tumor cells to increase ROS production [45].

Taking into account the significant effect of 3BrP in mitochondrial function we wondered if this might impact cell energy status. To address this issue, adenine nucleotides were quantified by High-performance liquid chromatography (HPLC; Fig 2D). In accordance with previous data, a tendency for a decrease in ATP levels in cells treated with 50 μM 3BrP was noted, although it was not significant. The same cannot be said about the control without LIF, where we observed a significant increase in ATP levels, consistent with differentiation (P<0.05). No differences were detected for ADP concentration while AMP levels were significantly increased in cells grown without LIF or in the presence of 50 μM 3BrP and LIF (P<0.01 and P<0.05 respectively). When considering more integrated measurements of energy charge (Fig 2E), we determined that our experimental conditions are inducing a lower energy charge in mESC when compared to the control, with the most significant decrease observed for cells without LIF or exposed to 50 μM 3BrP in the presence of LIF (P<0.05). When we take into account the AMP/ATP ratio (Fig 2F) there is a significant increase in cells without LIF and exposed to 50 μM 3BrP in the presence of LIF (P<0.01 and P<0.05, respectively), reflecting the differences in nucleotide concentrations. Furthermore the MTT assay was also used to indirectly infer metabolic activity, given that the assay measures cellular NADPH and NADH dehydrogenase activity and was normalized to total cell number. Our results demonstrate (Fig 2C) that 3BrP at a higher concentration negatively affects the metabolic activity of mESCs (P<0.05) and, again, that differentiated cells are more active when compared to our control condition (P<0.01). In short, although the effects of 3BrP seem consistent with the triggering of differentiation under pluripotency conditions (i.e. in the presence of LIF), these effects are clearly not due solely to a standard differentiation (removal of LIF in control colonies), as seen when comparing ROS, MTT and ATP levels for both conditions.

### Cell death in the presence of 3BrP

**Fig 3:**
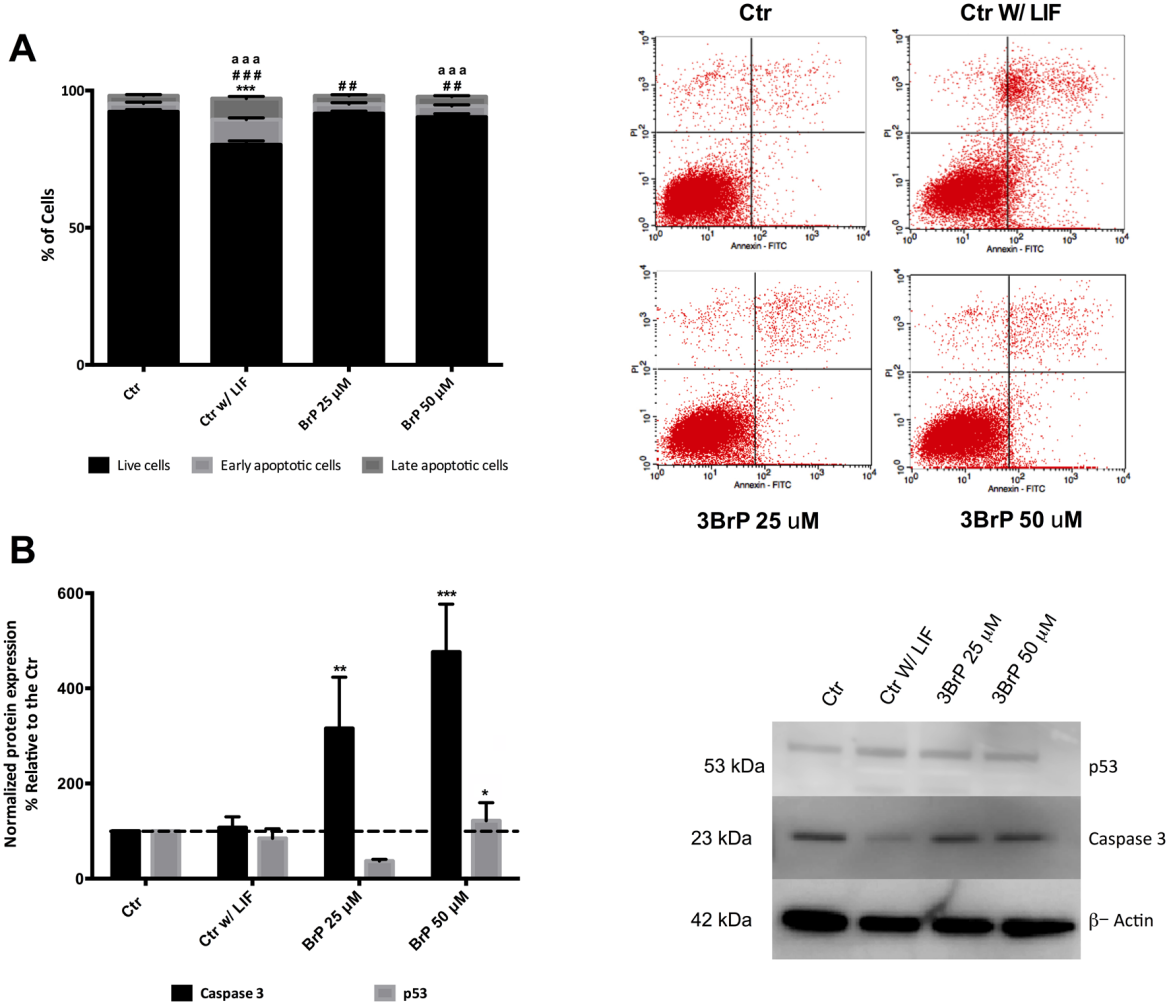
Assessment of apoptosis in mESC as affect by 3BrP. ESCs were maintained in control conditions (with LIF), in the absence of LIF (differentiation control) and in the presence of two different 3BrP concentrations (25 and 50 μM) plus LIF. **A)**- Apoptosis/ Necrosis was accessed by flow cytometry for Annexin v/PI and three populations were identified live cells (negative for both annexin V and PI); early apoptotic cells (positive for annexin V and negative for PI); late apoptotic cells (positive for both annexin V and PI). At the left side of the panel representative dot plots for all experimental conditions are presented. 20000 cells were evaluated for Geometric Mean of Fluorescence in each population. 4 independent experiments were performed. Symbols are used to compare levels of live (*), early apoptotic (#) and late apoptotic (a) cells. One, two and three symbols represent p≤ 0.05, p≤ 0.01, p≤ 0.001, respectively. **B)**- Western Blot quantification by densiometric evaluation for p53 and caspase3. Results are presented as percentage relative to the CTR and were normalized for beta-actin. 3 independent experiments were performed. *p≤ 0.05, **p≤ 0.01, ***p≤ 0.001.

Considering that 3BrP is a known apoptosis inducer, and even though we did not see significant differences in terms of cell viability, we performed AnnexinV/PI analysis by flow cytometry (Fig 3A). Notably, the absence of LIF drastically impacts cell survival (P<0.001), and a higher concentration of 3BrP once more is shown to be detrimental, while 25 μM 3BrP only presented significant differences for early apoptosis. Surprisingly only the experimental conditions with 3BrP showed significant changes in caspase 3 protein levels, with 3BrP 25 μM showing a significant increase in caspase 3 levels (P<0.01) and 50 μM 3BrP significantly increasing both p53 (P<0.001) and caspase 3 (P<0.001; Fig 3B).

### 3BrP effects on glycolytic enzymes and on mESC differentiation

**Fig 4:**
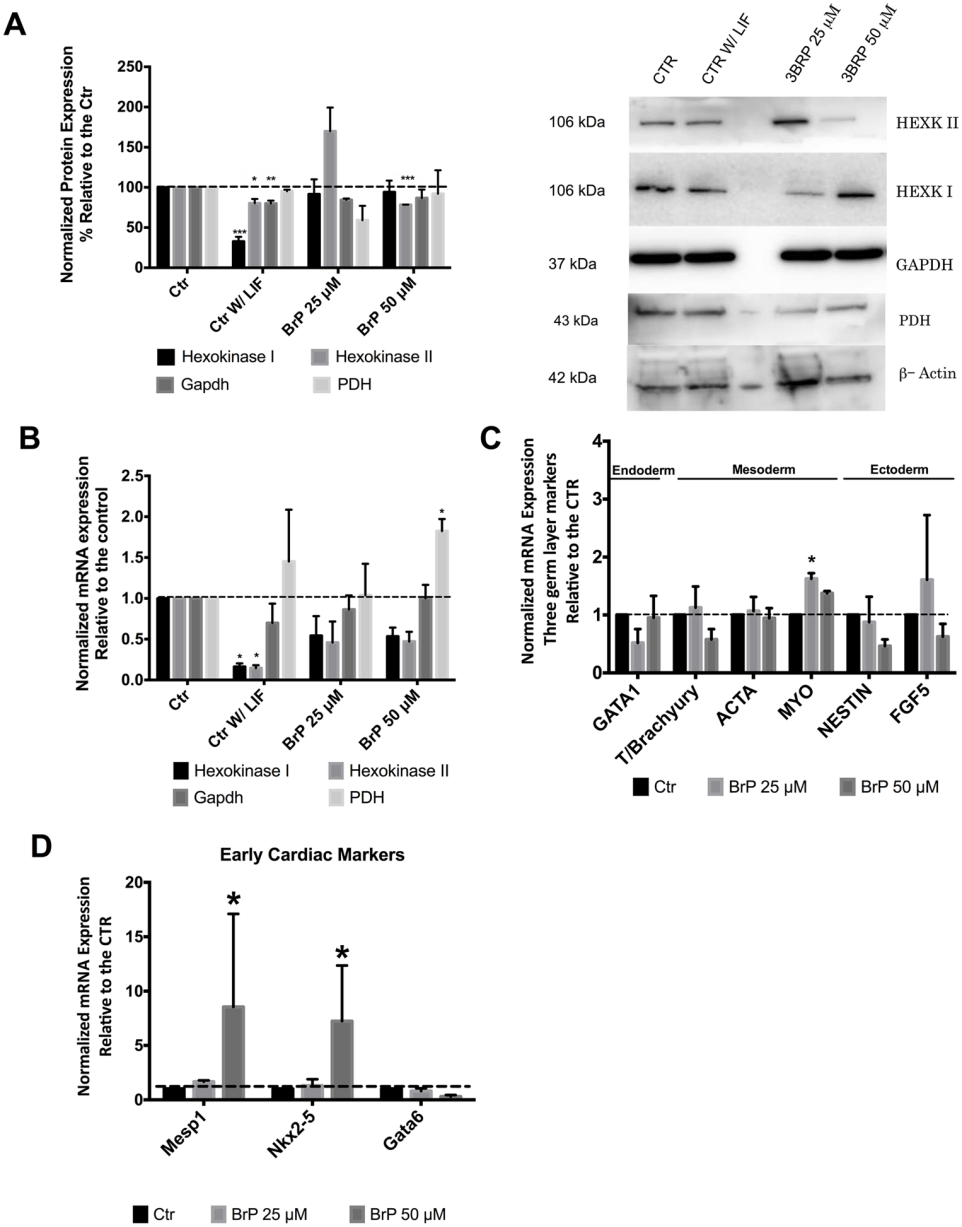
3BrP effect on glycolytic enzymes as well as on differentiation markers assessed via embryoid body (EB) formation. ESCs were maintained in control conditions (with LIF), in the absence of LIF (differentiation control) and in the presence of two different 3BrP concentrations (25 and 50 μM) plus LIF. **A)**- Left: Protein levels for Hexokinases II and I, Gapdh and PDH were determined by Western Blot and quantified by densiometric evaluation. Results were normalized to beta-Actin protein levels and are represented as percentage relative to the control; the results represent n = 4. Right: a representative blot demonstrating the decrease in protein levels for glycolytic enzymes. Molecular weight markers were run in lane 3, and some may have reacted with the secondary antibody. **B)**- RT-PCR analysis for Hexokinase I, II, Gapdh and PDH mRNA. Results are presented as fold changes and normalized to beta-Actin levels. 4 independent experiments were analyzed. **C)**- RT-PCR analysis for the mesoderm, endoderm and ectoderm germ layers markers following ESC differentiation in EBs. Results are presented as fold changes for 3 independent experiments, normalized to the reference house keeping gene beta-Actin. **D)**- RT-PCR analysis for mesoderm cardiac markers: Mesp1; Nkx2-5 and Gata6 following ESC differentiation in EBs. Results are presented as fold changes for 3 independent experiments, normalized to the reference house keeping gene beta-Actin. *p≤ 0.05, **p≤ 0.01, ***p≤ 0.001.

3BrP has been described to inhibit Hexokinase II (HKII) and, more recently, Gapdh [22]. Thus we examined protein and mRNA levels for some glycolytic enzymes in order to determine possible 3BrP effects on glycolysis. Interestingly, Western Blot analysis revealed that differentiating cells (control without LIF) presented more significant alterations in those enzymes when compared to the 3BrP effect (Fig 4A). For HKI we observed a significant decrease in our negative control both for protein (P<0.001) and mRNA levels (P<0.05; Fig 4B) while HKII protein levels were significantly decreased in Control without LIF (P<0.05) and 50 μM 3BrP (P<0.001) conditions. On the other hand, mRNA levels for both isoforms of Hexokinase decreased in the absence of LIF (P<0.05; Fig 4B). On the other hand Gapdh levels were not significantly affected by 3BrP in our experiments, the only effect noted was for differentiating cells in the absence of LIF, and only at the protein level. Furthermore no differences were observed in Pyruvate dehydrogenase (PDH) protein levels, which controls the fate of pyruvate conversion to either lactate or acetyl-CoA. However the same was not true for PDH mRNA levels, given that for the higher concentration of 3BrP there was a significant increase (P<0.05).

To determine if 3BrP could influence ESC differentiation we used the embryoid body assay, where cells are left to differentiate spontaneously by removing LIF, in this case in the presence or absence of 3BrP. The first thing to point out is the fact that control cells presented markers for the three germ layers upon differentiation, clearly demonstrating that ESCs were indeed pluripotent (Fig 4C). Given that initially the only significant difference was observed for the mesoderm marker MyoD, which is a marker for muscle development, we decided to analyze other markers focusing exclusively on early cardiac muscle markers, such as Mesp1, Nkx2-5 and Gata6, given that cardiac differentiation implies an increase in mitochondrial function (Fig 4D). Overall we found three markers differentially expressed, suggesting that 3BrP could possibly lead to a bias differentiation towards mesoderm, particularly cardiac development.

## Discussion

Embryonic stem cells (ESC) in general rely more on glycolysis and a shift to a predominantly oxidative metabolism (OXPHOS) is though to be needed for differentiation to take place [7]. Due to the metabolic characteristics of cancer cells novel therapeutic approaches have focused on inhibiting glycolysis, and 3BrP has become a promising compound in this respect, because, although it also has other cellular targets, it inhibits glycolytic enzymes and alters mitochondrial function. Indeed promising results regarding decrease in tumor size and cell death have been noted with this compound [24, 43, 46].

Considering the metabolic parallelism between ESC and cancer cells [7], we wondered weather 3BrP would affect pluripotent ESCs in the same manner. In order to address this issue we treated cells with 3BrP (concentrations in the range used in cancer research—0–80 mM [43]) in the presence of LIF, and monitored changes in mESC pluripotency to determine if 3BrP would induce metabolic changes that might shift cells towards differentiation, even under stringent pluripotency conditions. To our knowledge this is the first paper describing such an experimental approach for ESCs. Titrations were performed in order to determine the lowest concentration of 3BrP needed to affect ESC, and we chose as a negative control cells grown without LIF (i.e. differentiating cells).

We show that 3BrP has a negative impact on colony morphology, cell number, viability and cell proliferation, although not as negative as growing ESCs without LIF. Overall, in the presence of 3BrP colonies were smaller and borders started to lose definition. These observations are paralleled by significant differences in total cell number, although with no detrimental effects on viability, suggesting that 3BrP affects cell proliferation rates. The effects of 3BrP on cell viability are well described, as this compound causes both apoptosis and necrosis by depleting ATP due to impairment of mitochondrial function [47, 48]. The reason we did not observe such an effect in our Live/Dead assay could be due to PI only staining cells with compromised membranes, which is a very late phenomenon in cell death. This was further confirmed with our annexinV/PI results, which revealed a negative impact of 3BrP for all experimental conditions. These results were supported by the Western blot analysis of p53 and caspase3 protein levels and point to a possible stressful cellular condition, which for differentiating cells is probably a normal process of cell selection, while in the case of 3BrP we cannot rule out a toxic effect, which could explain the significant decrease in total cell number.

Although 3BrP is possibly causing toxic effects we could not rule out an additional impact in the cell cycle. Both pluripotent stem cells and certain cancer cells proliferate extensively, and thus require bioenergetic and biosynthetic blocks for cell growth and division [7]. Given the observed impact in total cell number, we analyzed PCNA, which is involved in DNA synthesis during replication and can interact with different cell cycle proteins including p21 [49, 50]. It is also important to note that PCNA staining allowed us to distinguish a “true” differentiation effect (lack of LIF) from a 3BrP effect. However 3BrP did lead to an apparent loss of pluripotency as analyzed by protein and mRNA levels for Oct4 (and Nanog, to a lesser extent and only at the protein level), as well as the significant decrease in AP-positive colonies. It should be noted that the results observed for cells grown without LIF are similar to the ones obtained with cells exposed to 50 μM 3BrP, showing that the highest 3BrP concentration is more detrimental for pluripotency.

As discussed previously, 3BrP affects mitochondrial function in cancer cells, so we investigated mitochondrial-related changes in ESC in the presence of this compound. Overall mitochondria become more active, reflected by the significant increase in MMP following 3BrP treatment, accompanied by an increase in the production of superoxide anion. For the higher concentration of 3BrP energy charge decreased, a reflection from the significant increase in AMP level and the tendency for a decrease in ATP levels, which could mean that the increment in MMP and ROS production is starting to be detrimental to mitochondrial function. MTT results also suggest a slower metabolic state of cells exposed to 3BrP, and given that MTT measures dehydrogenase activity treated cells could have a lower capability to manage oxidative stress as well. This outcome is characteristic of 3BrP. On the other hand, as with PCNA results, cells grown without LIF were slightly different, with higher concentrations of ATP and AMP, and a significant increase in MTT, thus suggesting that these cells are more active and capable of managing oxidative stress.

Besides a detrimental effect on mitochondrial function and apoptosis effect 3BrP could affect metabolic enzymes promoting a metabolic shift that would result in the loss of pluripotency. In order to address this question we quantified protein and mRNA levels for Hexokinase I and II, as well as other downstream glycolytic enzymes. Although results were not linear, they suggest a decrease in both Hexokinase isoforms and Gapdh (both purported targets for 3BrP inhibition [22]), at the protein and mRNA levels upon differentiation. 3BrP seems to negatively impact Hexokinases in particular HKII at the protein level, which could lead to a shift in metabolism; however, more experiments are needed before definitive conclusions can be drawn. Notably, any conclusions on more specific roles for Hexokinases and Gapdh in pluripotency would certainly require molecular-based assays, such as enzyme overexpression/downregulation, which, as stated previously, was not the purpose of the present work.

Overall our results suggest that by using 3BrP we are promoting pluripotency loss even under pluripotency culture conditions, and possibly a shift in metabolism, considering that mitochondria are more active. However, the effects of 3BrP in the presence of LIF do not always mirror what takes place under normal differentiation conditions (absence of LIF), suggesting that 3BrP is not simply shifting ESCs towards differentiation, but also causing other effects, and thus that the parallelism between stemness and cancer must be treated with caution. In addition, differentiation seems to be accompanied by a decrease in Gapdh and Hif 1α levels, which is very interesting given that Hif-1α participates in the maintenance of stem cell identity by interacting with core pluripotency transcriptional factors, and also increases the expression of glycolytic enzymes [7, 8, 16].

Although embryonic stem cells (ESC) rely more on glycolysis it is important to note that there may be a metabolically bivalent metabolic state early in cell commitment [51]. Regardless the first main conclusion of this study is that the putative chemotherapeutic agent 3BrP affects ESC pluripotency, but does not trigger forced differentiation in the same manner as removing LIF. The second goal of the study was to determine if the compound would hasten triggered ESC differentiation, or create a useful differentiation bias. Indeed, 3BrP seemed to induce biased differentiation, given that differentiation in the presence of the compound was associated with predominance in cardiac precursors markers. However, this bias was not of a magnitude that would definitely imply a clear practical use in this case.

## Supporting Information

S1 Fig Representative Dot-Plots and Histograms for flow cytometry experiments. ESCs were maintained in control conditions (with LIF), in the absence of LIF (differentiation control) and in the presence of two different 3BrP concentrations (25 and 50 μM) plus LIF. Dot-Plots and overlay of Histograms for analysis of: **(A)** intracellular amounts of superoxide using MitoSOX Red (Molecular Probes); **(B)** evaluation of the expression of the proliferating cell nuclear antigen (PCNA) and **(C)** mitochondrial membrane potential using TMRM (Invitrogen). For MitoSOX Red and PCNA experimental conditions were: Control (upper left panel); Control without LIF (upper right); 3BrP 25 μM+LIF (lower left) and 3BrP 50 μM + LIF (lower right). For TMRM analysis the Dot-Plots are: Control (upper left panel); Control without LIF (upper central panel); Control without LIF for 5 days (upper right panel); 3BrP 25 μM+ LIF (lower left panel) and 3BrP 50 μM + LIF (lower right panel). The histograms show representative experiments in an overlay display in order to better represent the data.
(TIF)

S1 Table List and sequence of primers obtained from the primer bank database http://pga.mgh.harvard.edu/primerbank/. Primers were used for the genes listed as described in the text.
(DOCX)
