# Supplementary figures and images for: Differentiate or Die: 3-Bromopyruvate and Pluripotency in Mouse Embryonic Stem Cells
Source: PLoS One. 2015 Aug 12;10(8):e0135617. doi: 10.1371/journal.pone.0135617 (PMC4534445; doi:10.1371/journal.pone.0135617)

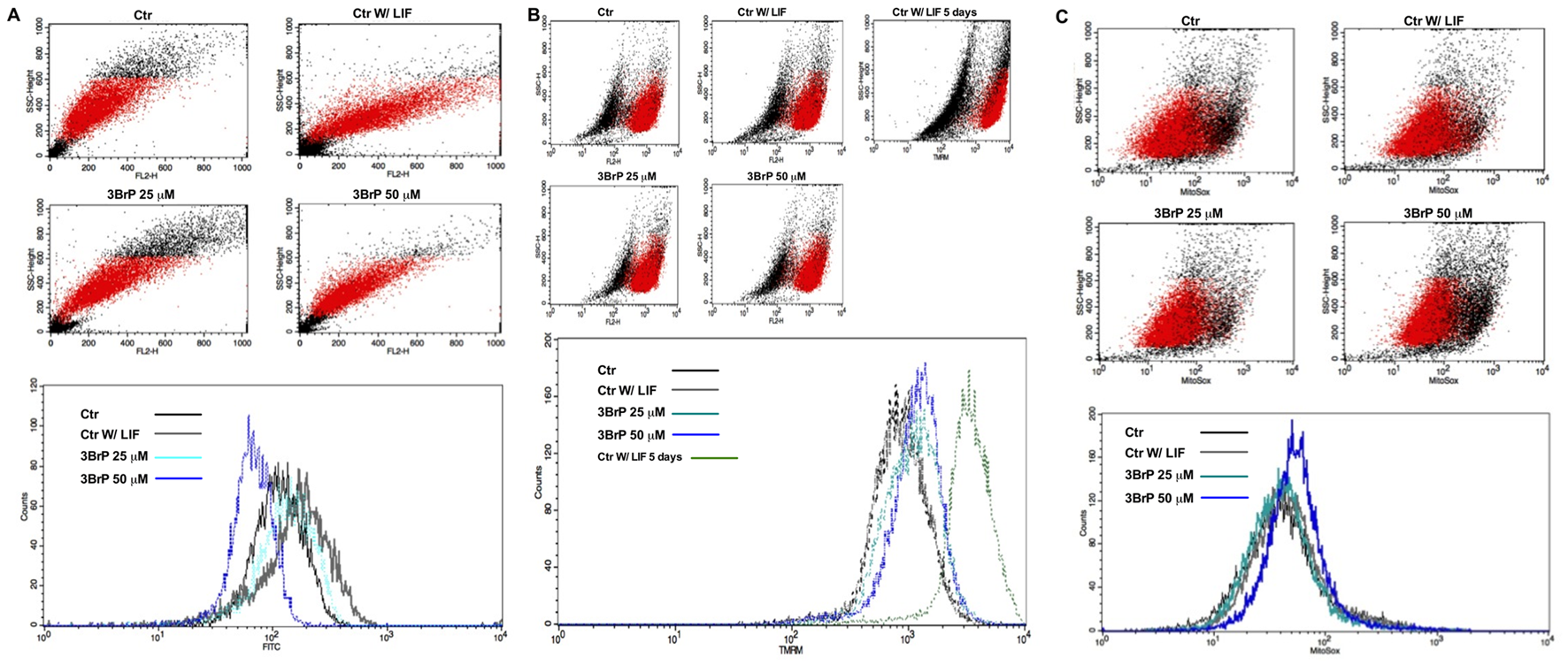

Supplement: S1 Fig — ESCs were maintained in control conditions (with LIF), in the absence of LIF (differentiation control) and in the presence of two different 3BrP concentrations (25 and 50 μM) plus LIF. Dot-Plots and overlay of Histograms for analysis of: (A) intracellular amounts of superoxide using MitoSOX Red (Molecular Probes); (B) evaluation of the expression of the proliferating cell nuclear antigen (PCNA) and (C) mitochondrial membrane potential using TMRM (Invitrogen). For MitoSOX Red and PCNA experimental conditions were: Control (upper left panel); Control without LIF (upper right); 3BrP 25 μM+LIF (lower left) and 3BrP 50 μM + LIF (lower right). For TMRM analysis the Dot-Plots are: Control (upper left panel); Control without LIF (upper central panel); Control without LIF for 5 days (upper right panel); 3BrP 25 μM+ LIF (lower left panel) and 3BrP 50 μM + LIF (lower right panel). The histograms show representative experiments in an overlay display in order to better represent the data. (TIF) [file pone.0135617.s001.tif]
