# Supplementary material for: Differentiate or Die: 3-Bromopyruvate and Pluripotency in Mouse Embryonic Stem Cells
Source: PLoS One. 2015 Aug 12;10(8):e0135617. doi: 10.1371/journal.pone.0135617 (PMC4534445; doi:10.1371/journal.pone.0135617)
Supplement: S1 Table — Primers were used for the genes listed as described in the text. (DOCX) [file pone.0135617.s002.docx]

**S1 Table. List and sequence of primers obtained from the primer bank database** [**http://pga.mgh.harvard.edu/primerbank/**](http://pga.mgh.harvard.edu/primerbank/)**:** Primers were used for the genes listed as described in the text.

| ***Gene*** |  | ***Sequence*** | ***Gene Bank ID*** |
| --- | --- | --- | --- |
| ***Oct4*** | Fw: | CGGAAGAGAAAGCGAACTAGC | 356995852c3 |
|  | RV: | ATTGGCGATGTGAGTGATCTG |  |
| ***Nanog*** | Fw: | TCTTCCTGGTCCCCACAGTTT | 31338864a1 |
|  | RV: | GCAAGAATAGTTCTCGGGATGAA |  |
| ***Beta-Actin*** | Fw: | GGCTGTATTCCCCTCCATCG | 6671509a1 |
|  | RV: | CCAGTTGGTAACAATGCCATGT |  |
| ***Gapdh*** | Fw: | AGGTCGGTGTGAACGGATTTG | 6679937a1 |
|  | RV: | TGTAGACCATGTAGTTGAGGTCA |  |
| ***Pdh*** | Fw: | GAAATGTGACCTTCATCGGCT | 6679261a1 |
|  | RV: | TGATCCGCCTTTAGCTCCATC |  |
| ***Hexokinase II*** | Fw: | TGATCGCCTGCTTATTCACGG | 7305143a1 |
|  | RV: | AACCGCCTAGAAATCTCCAGA |  |
| ***Hexokinase I*** | Fw: | CGGAATGGGGAGCCTTTGG | 309289a1 |
|  | RV: | GCCTTCCTTATCCGTTTCAATGG |  |
| ***Fgf5*** | Fw: | AAGTAGCGCGACGTTTTCTTC | 3721900a1 |
|  | RV: | CTGGAAACTGCTATGTTCCGAG |  |
| ***Mesp1*** | Fw: | GTCACTCGGTCCTGGTTTAAG | 33469091a1 |
|  | RV: | ACGATGGGTCCCACGATTCT |  |
| ***Myod1*** | Fw: | CCACTCCGGGACATAGACTTG | 6996932a1 |
|  | RV: | AAAAGCGCAGGTCTGGTGAG |  |
| ***T (Brachyury)*** | Fw: | GCTTCAAGGAGCTAACTAACGAG | 6678203a1 |
|  | RV: | CCAGCAAGAAAGAGTACATGGC |  |
| ***Gata1*** | Fw: | TGGGGACCTCAGAACCCTTG | 6679947a1 |
|  | RV: | GGCTGCATTTGGGGAAGTG |  |
| ***Gata6*** | Fw: | TTGCTCCGGTAACAGCAGTG | 33859556a1 |
|  | RV: | GTGGTCGCTTGTGTAGAAGGA |  |
| ***Nkx2-5*** | Fw: | GACAAAGCCGAGACGGATGG | 6679068a1 |
|  | RV: | CTGTCGCTTGCACTTGTAGC |  |
| ***Acta2*** | Fw: | GTCCCAGACATCAGGGAGTAA | 6671507a1 |
|  | RV: | TCGGATACTTCAGCGTCAGGA |  |
| ***Nestin*** | Fw: | CCCTGAAGTCGAGGAGCTG | 15011851a1 |
|  | RV: | CTGCTGCACCTCTAAGCGA |  |
